# Effect of prednisone, aspirin, low molecular weight heparin and intravenous immunoglobulin on outcome of pregnancy in women with antiphospholipid syndrome

**DOI:** 10.3892/etm.2012.743

**Published:** 2012-10-10

**Authors:** JING XIAO, JING XIONG, FUFAN ZHU, LIANG HE

**Affiliations:** Department of Gynecology and Obstetrics, The Second Xiangya Hospital, Central South University, Changsha, Hunan 410011, P.R. China

**Keywords:** antiphospholipid syndrome, live birth, intravenous immunoglobulin, obstetric complication, low molecular weight heparin

## Abstract

The aim of this study was to evaluate the effect of traditional treatment (prednisone and aspirin) and comprehensive treatment [prednisone, aspirin, low molecular weight heparin (LMWH) and IVIg] on the pregnancy outcome, obstetric complications and fetal outcome in women with antiphospholipid syndrome (APS). In the present trial, we observed and evaluated 129 women with APS. Eighty-seven patients received traditional treatment and 42 patients received comprehensive treatment. In the traditional treatment group and comprehensive treatment group, the live birth rate was 83.91 and 97.62% (P<0.05), respectively, and the obstetric morbidity was 22.99 and 7.14% (P<0.05), respectively. The neonatal weight in the comprehensive treatment group was increased compared with the traditional treatment group (P<0.05), however, no differences were found in gestational age at delivery or preterm labor. Comprehensive treatment improved the result of gestation and reduced obstetric complications, and is a more effective treatment for APS than the traditional method using prednisone and aspirin.

## Introduction

Antiphospholipid syndrome (APS) is an autoimmune disease characterized by recurrent arterial and/or venous thrombosis, recurrent fetal loss and positive antiphospholipid (aPL) antibodies. Pregnancy complications are common in patients with APS and mainly include fetal growth restriction (FGR), preeclampsia and preterm labor (1). Recently proposed mechanisms include antibody-mediated interference with coagulation homeostasis, platelet activation, endothelial cell activation, placental tissue injury, T-cell immune response and complement activation (2,3).

Various therapies have been used clinically to treat APS, alone and in combination, but there is no general standard and therapy remains challenging. The traditional treatment with prednisone and aspirin, which has been used since the 1980s for immunosuppression and anticoagulation, is efficient (4), but may induce obstetric complications, particularly preterm delivery and preeclampsia. The combination of heparin, particularly low molecular weight heparin (LMWH) and aspirin is considered superior to prednisone and aspirin, not because it achieves higher live birth rates, but because it causes less maternal morbidity (5). Intravenous immunoglobulin (IVIg) contains anti-idiotypic antibodies, has an immunomodulatory action and may be used to treat autoimmune diseases. There are numerous case reports of successful pregnancy outcomes after treatment with IVIg (6–8).

There are no definitive conclusions regarding the safety of drugs during pregnancy. Prednisone, aspirin, LMWH and IVIg each have advantages and disadvantages for treating APS. Long-term usage of prednisone during pregnancy may provoke obstetric complications. However, it is efficient, inexpensive and convenient to use and may be one of the best choices for non-pregnant patients with APS. LMWH is safe and effective, but long-term subcutaneous injection causes pain and inconvenience. IVIg therapy is too expensive for widespread clinical use, and there is a lack of large clinical trials.

The present clinical comparative study aimed to evaluate the effect of treatment with prednisone, aspirin, LMWH and IVIg. APS patients were observed from the time of positive pregnancy testing until delivery or miscarriage. To the best of our knowledge, this study is the first randomized trial to evaluate the effect of comprehensive treatment with prednisone, aspirin, LMWH and IVIg in patients with APS.

## Materials and methods

### Subjects

The patients who participated in this clinical comparative study were referred to the Department of Gynaecology and Obstetrics at the Second Xiangya Hospital of Central South University (Changsha, China) between March 2008 and December 2010. The study was approved by the ethics committee of the 2nd Xiangya Hospital of Central South University and all patients provided informed consent. APS was diagnosed using Sapporo diagnostic criteria. The first of these involved vascular thrombosis, one or more clinical episodes of arterial, venous or small vessel thrombosis were present in any tissue or organ. Thrombosis must be confirmed by objective validated criteria (i.e., unequivocal findings of appropriate imaging studies or histopathology). For histopathological confirmation, the thrombosis should be present without significant evidence of inflammation in the vessel wall. The second criterion pertained to pregnancy morbidity including i) one or more unexplained deaths of a morphologically normal fetus at or beyond the 10th week of gestation, with normal fetal morphology documented by ultrasound or by direct examination of the fetus, or ii) one or more premature births of a morphologically normal neonate prior to the 34th week of gestation due to eclampsia or severe preeclampsia defined according to standard definitions, or recognized features of placental insufficiency, or iii) three or more unexplained consecutive spontaneous abortions prior to the 10th week of gestation, with maternal anatomical or hormonal abnormalities and paternal and maternal chromosomal causes excluded. In studies of populations of patients with more than one type of pregnancy morbidity, investigators were strongly encouraged to stratify groups of subjects according to i, ii or iii above. The third criterion involved the following laboratory criteria: a) Lupus anticoagulant (LA) present in plasma, on two or more occasions at least 12 weeks apart, detected according to the guidelines of the International Society on Thrombosis and Haemostasis (1); b) anticardiolipin (aCL) antibody of IgG and/or IgM isotype in serum or plasma, present in medium or high titer (i.e., >40 GPL or MPL, or > the 99th percentile), on two or more occasions, at least 12 weeks apart, measured by a standardized ELISA; c) anti-β2 glycoprotein-I antibody of IgG and/or IgM isotype in serum or plasma (in titer > the 99th percentile), present on two or more occasions, at least 12 weeks apart, measured by a standardized ELISA, according to recommended procedures.

The entry criteria were patients who wanted to have a baby, agreed to a complete evaluation, had at least two documented pregnancy losses, were positive for aPL antibody on at least two occasions, and agreed to be assigned to prednisone and aspirin or prednisone and aspirin plus LMWH and IVIg. Exclusion criteria were patients with chromosomal abnormality, endocrine secretion disease, blood type incompatibility, infectious disease, genital tract malformation, systemic lupus erythematosus and any other immune diseases.

In total, 196 patients were investigated and randomized into two groups. Of these, 26 patients were lost to follow-up and 41 left the study, ultimately only 129 patients were analyzed. Of the 129 patients, 87 accepted the combination treatment of prednisone and aspirin, and 42 accepted the comprehensive therapy of prednisone, aspirin, LMWH and IVIg.

### Patient interviews and monitoring

Patients were given treatment as soon as a positive pregnancy test as identified by urine human choriogonadotropin testing at approximately 5 weeks of gestation was achieved. The subjects were tested each month for aPL antibodies. If aPL antibodies were negative, treatment was ceased. When aPL antibodies remained positive during pregnancy, the therapy was continued throughout the gestational period.

Pregnancy in the two groups was confirmed by two rising quantitative β-human chorionic gonadotropin hormone levels measured 48 h apart, or by ultrasound confirmation of fetal heart activity at 7–8 weeks. The patients were evaluated every 2–4 weeks during pregnancy. The patients were interviewed, and medical records were reviewed to confirm pregnancy histories. Prenatal diagnosis was performed at 8–20 weeks. Multicolor ultrasonography was conducted at 24–28 weeks and performed serially to assess fetal growth and amniotic fluid volume every 4 weeks. Uterine and umbilical artery blood velocity waveforms were assessed every 2–4 weeks from 28 weeks. Weekly, non-stress testing was performed from 34 weeks. When complications were identified, the patient was provided with the appropriate treatment.

### Drug usage

Prednisone was administered at a low dose of 5 mg three times per day. Aspirin was administered at a low dose of 50 mg once per day. LMWH (Dalteparin Sodium) was subcutaneously injected once daily in the anterior abdominal wall at a dose of 5000 IU. IVIg was administered intravenously at a dose of 10 g per day for five consecutive days in a month, repeated two or three times depending on the severity of the illness.

### Outcome assessment

A positive pregnancy outcome was the birth of a live infant, a negative pregnancy outcome was considered to be miscarriage, fetal death or stillbirth. An obstetric complication was classified as one of the following events: preeclampsia (systolic blood pressure >140 mmHg or diastolic blood pressure >90 mmHg associated with proteinuria >300 mg/24 h), preterm labour (live birth before 37 weeks of gestation), FGR (birth weight <10th percentile of the standard growth curve), premature rupture of membrane (PROM; rupture of membrane prior to labour), gestational diabetes mellitus (GDM; diagnosed by 2 h plasma glucose after dinner ≥7.8 mmol/l or oral glucose tolerance test abnormal). For fetal outcome, we recorded the live birth ratio, gestational age at delivery, preterm labour ratio, and neonatal weight.

### Statistical analysis

Statistical analysis was performed using SPSS 17.0 (Chicago, IL, USA). Quantitative variables were analysed using Student’s t-test and qualitative variables using the Chi-square test. P<0.05 was considered to indicate a statistically significant result.

## Results

### Patient characteristics

All 129 patients enrolled in the study had a history of miscarriage before 12 weeks gestational age. The age of the patients ranged from 22 to 36 years old. There were no significant differences in patient age, total number of prior pregnancies, prior live births or miscarriages between the two groups (Table I). Each patient had a positive result for aPL antibody testing.

### Pregnancy outcome

Of the 129 patients, 114 had live births. Mothers and infants were all in good health. Twelve patients had an abortion before 10 weeks, three patients had fetal death and none had stillbirth. The live birth ratio was 88.37% (114/129), and the fetal loss rate was 11.63% (15/129).

Of the 87 patients treated with prednisone and aspirin, 73 had live births, 11 had abortions prior to 10 weeks, three had fetal deaths and none had stillbirths. The live birth ratio was 83.91% (73/87) and the fetal loss rate was 16.09% (14/87).

Of the 42 patients treated with prednisone and aspirin plus LMWH and IVIg, 41 had live births. Only one woman had an abortion. The live birth ratio was 97.62% (41/42) and the fetal loss rate was 2.38% (1/42).

As shown in Table II, the difference in the number of live births between the two groups was significant (P<0.05).

### Obstetric complications

Of the 129 patients, 20 had pregnancy complications, of which 17 had one complication, two had premature labor and preeclampsia, and one had premature labor and PROM. All the complications included premature labour in nine cases, FGR in four cases, preeclampsia in three cases, PROM in four cases and GDM in three cases. The total rate of pregnancy complications was 17.83%. In the traditional treatment group, 20 cases had obstetric complications, with the obstetric morbidity being 22.99%. In the comprehensive treatment group, three cases had obstetric complications, with the obstetric morbidity being 7.14%. The difference in the incidence of complications between the two treatment groups was statistically significant (P<0.05) (Table III).

There were no differences in preterm births, FGR, preeclampsia or GDM. There were no episodes of major bleeding or fetal malformation in either treatment group (data not shown).

### Fetal outcome

To assess fetal outcome, gestational age was recorded at delivery, as well as the preterm labour ratio and neonatal weight. Neonatal weight in the comprehensive treatment group was significantly increased compared with the traditional treatment group (P<0.05), but there were no differences in gestational age at delivery or preterm labour (Table IV).

## Discussion

An ideal treatment for APS would: i) prevent pregnancy loss and increase live births; ii) improve maternal and fetal/neonatal outcome by preventing maternal morbidity, including preeclampsia, preterm birth and placental insufficiency; iii) reduce or eliminate aPL antibodies that induce the disease.

Several regimens have been proposed for the treatment of APS, including aspirin alone, combination of prednisone and aspirin, LMWH and prednisone, and more recently, IVIg. Prednisone, a short-acting corticosteroid, is a potent anti-inflammatory drug. Low to moderate doses of prednisone are safe throughout pregnancy, but may increase the incidence of PROM and hypertension. Low-dose aspirin, used as an antiplatelet agent, appears to be safe throughout pregnancy; however, histological studies have shown that intravascular or intervillous blood clots are not commonly observed in miscarriage samples from APS patients (9). Although numerous studies have suggested that prednisone and/or aspirin alone or in combination during pregnancy may improve pregnancy outcome by 75–100% (4,5), recent studies indicate that LMWH and IVIg may be more efficient (10,11).

LMWH does not cross the placenta and is safe for both mother and fetus. Various clinical studies have demonstrated its function in improving live birth rates in patients with APS (10,11). However, the mechanism by which it reduces pregnancy loss remains unclear. LMWH may play a role via anticoagulation; blocking activation of complement C3 and C5 by aPL antibodies targeted at decidual tissue (12); immunomodulating cell-mediated or humoral events by influencing pro-inflammatory and anti-inflammatory cytokine generation (13); implantation and trophoblastic invasion by down-regulation of decidual E-selectin expression and modulation of selectin-mediated cell adhesion (14); and reducing the ability of aPL antibodies to recognize phospholipids (15). However, osteoporosis and fractures are reported in patients treated with LMWH during pregnancy.

Use of IVIg to prevent recurrent pregnancy loss associated with APS was first reported in 1988. Previous studies have suggested that IVIg is superior to prednisone and aspirin for the treatment of APS (6). IVIg is able to: neutralize pathogenic auto-antibodies; inhibit the action of pathological antibodies by the interaction of the Fc portion of immunoglobulin with Fc or Fab receptors, or by passively acting as anti-idiotypic antibodies; block binding of the autoantibody at the level of the endothelial cell, thereby eliminating the thrombogenic effects of aPL antibodies and the activation of endothelial cells (7); and adjust production of pro-inflammatory and anti-inflammatory cytokines, and interact with complement activation. A study in 1999 suggesting that IVIg was to be added to the standard therapy resulted in excellent fetal and maternal outcome, although its definitive role awaits the results of randomised trials (8).

This trial demonstrated that comprehensive treatment with prednisone and aspirin plus LMWH and IVIg may be superior to the traditional method with prednisone and aspirin in the treatment of APS. The comprehensive treatment not only leads to a significantly higher rate of live births, but also has fewer obstetric complications than prednisone and aspirin in patients with a history of recurrent miscarriage associated with aPL antibodies.

The trial was not blinded since it was not considered ethical to give daily subcutaneous injections of a placebo to a pregnant patient. However, to the best of our knowledge, this study is the first randomized trial to evaluate the effect of comprehensive treatment with prednisone, aspirin, LMWH and IVIg in patients with APS. We observed that the 42 women who were treated with prednisone and aspirin plus LMWH and IVIg had an improved live birth rate compared with the 87 patients who were treated with prednisone and aspirin. In APS, the most significant problem is recurrent miscarriage; however, the mechanisms are complex. Comprehensive treatment with LMWH and IVIg together with traditional treatment of prednisone and aspirin has multiple functions, including anti-inflammatory, antiplatelet, anticoagulation, blocking activation of complement, immunomodulation, regulating the function of endothelial cells and promoting implantation and trophoblastic invasion. Comprehensive treatment almost entirely blocks the pathogenesis of APS, thereby achieving an improved live birth rate (the live birth rate in the traditional treatment group was 83.91% and in the comprehensive treatment group was 97.62%) and improved fetal outcome.

In addition, due to its multiple functions, the placental microcirculation, shallow placental implantation and placental injury in APS patients may be improved with this treatment, leading to fewer obstetric complications. Our data indicate that LMWH and IVIg treatment of APS resulted in a significant decrease in obstetric morbidity. However, the obstetric complication rate was low in both treatment groups (22.99% with traditional treatment and 7.14% with comprehensive treatment) since we did not include any normal pregnancies nor untreated pregnancies with APS in this trial; thus, it cannot be concluded that traditional treatment was associated with more obstetric complications.

In the present study, we found the abortion rate was much lower than the natural abortion rate. This difference may be due to the inclusion criteria, since numerous diseases were excluded at the beginning of the study.

In conclusion, the data reported here indicate that comprehensive treatment with prednisone, aspirin, LMWH and IVIg is a safe and effective therapy for patients with APS, improving the result of gestation, as well as reducing maternal and obstetric complications.

## Figures and Tables

**Table I:** Patient characteristics.

Variable	Age	No. of prior pregnancies	No. of prior live births	No. of miscarriages
Traditional treatment, n=87 (%)	28.93±4.63	2.89±1.18	0.25±0.46	2.20±0.43
Comprehensive treatment, n=42 (%)	27.92±3.90	3.05±0.96	0.20±0.40	2.24±0.48
P-value	NS	NS	NS	NS

The traditional treatment group was provided with prednisone plus aspirin. The comprehensive treatment group was provided with prednisone and aspirin plus low molecular weight heparin and intravenous immunoglobulin. Quantitative variables were analyzed using the Student’s t-test; P<0.05 was considered to indicate a statistically significant result. NS, not significant.

**Table II:** Patient outcome data.

Variable	Traditional treatment n=87 (%)	Comprehensive treatment n=42 (%)	Total n=129 (%)	P-value
Live birth	73 (83.91)	41 (97.62)	114 (88.37)	0.011
Fetal loss	14 (16.09)	1 (2.38)	15 (11.63)	
Miscarriage	11	1	12	
Fetal death	3	0	3	
Stillbirth	0	0	0	

The traditional treatment group was provided with prednisone plus aspirin. The comprehensive treatment group was provided with prednisone and aspirin plus low molecular weight heparin and intravenous immunoglobulin. Qualitative variables were analyzed using the Chi-square test. P<0.05 was considered to indicate a statistically significant result.

**Table III:** Difference of obstetric complications between the two treatment groups.

Variable	Preterm labor	Preeclampsia	FGR	PROM	GDM	Total complications
Total, n=129 (%)	9 (6.98)	3 (2.33)	4 (3.10)	4 (3.10)	3 (2.33)	23 (17.38)
Traditional treatment, n=87 (%)	8 (9.20)	3 (3.45)	3 (3.45)	3 (3.45)	3 (3.45)	20 (22.99)
Comprehensive treatment, n=41 (%)	1 (2.38)	0 (0.00)	1 (2.38)	1 (2.38)	0 (0.00)	3 (7.14)
P-value	NS	NS	NS	NS	NS	0.028

The traditional treatment group was provided with prednisone plus aspirin. The comprehensive treatment group was provided with prednisone and aspirin plus low molecular weight heparin and intravenous immunoglobulin. Twenty women had pregnancy complications, of whom 17 had one complication, two had premature labor and preeclampsia, and one had premature labor and PROM. Qualitative variables were analyzed using the Chi-square test; P<0.05 was considered to indicate a statistically significant result. NS, not significant; FGR, fetal growth restriction; PROM, premature rupture of membrane; GDM, gestational diabetes mellitus.

**Table IV:** Fetal outcomes in live births.

Variable	Gestational age at delivery	Preterm labour	Neonatal weight
Traditional treatment, n=73 (%)	38.13±1.51	8 (10.96)	2842.17±316.47g
Comprehensive treatment, n=41 (%)	38.04±1.27	1 (2.44)	2987.14±245.26g
P-value	NS	NS	0.002

The traditional treatment group was provided with prednisone plus aspirin. The comprehensive treatment group was provided with prednisone and aspirin plus low molecular weight heparin and intravenous immunoglobulin. Quantitative variables were analyzed using the Student’s t-test, while qualitative variables were analyzed using the Chi-square test. P<0.05 was considered to indicate a statistically significant result. NS, not significant.
